# The accumulation of muscle RING finger-1 in regenerating myofibers: Implications for muscle repair in immune-mediated necrotizing myopathy

**DOI:** 10.3389/fneur.2022.1032738

**Published:** 2022-11-24

**Authors:** Meng-Ge Yang, Qing Zhang, Hong Wang, Xue Ma, Suqiong Ji, Yue Li, Li Xu, Zhuajin Bi, Bitao Bu

**Affiliations:** ^1^Department of Neurology, Tongji Hospital of Tongji Medical College, Huazhong University of Science and Technology, Wuhan, China; ^2^Genetic Diagnostic Centre, Department of Internal Medicine, Tongji Hospital of Tongji Medical College, Huazhong University of Science and Technology, Wuhan, China

**Keywords:** muscle RING finger-1, immune-mediated necrotizing myopathy, dermatomyositis, dysferlinopathy, regeneration

## Abstract

**Background:**

Muscle RING finger-1 (MuRF-1) plays a key role in the degradation of skeletal muscle proteins. We hypothesize the involvement of MuRF-1 in immune-mediated necrotizing myopathy (IMNM).

**Methods:**

Muscle biopsies from patients with IMNM (*n* = 37) were analyzed and compared to biopsies from patients with dermatomyositis (DM, *n* = 13), dysferlinopathy (*n* = 9) and controls (*n* = 7) using immunostaining.

**Results:**

MuRF-1 staining could be observed in IMNM, DM and dysferlinopathy biopsies, whereas the percentage of MuRF-1 positive myofibers was significantly higher in IMNM than in dysferlinopathy (*p* = 0.0448), and positively correlated with muscle weakness and disease activity in IMNM and DM. Surprisingly, MuRF-1 staining predominantly presented in regenerating fibers but not in atrophic fibers. Moreover, MuRF-1-positive fibers tended to be distributed around necrotic myofibers and myofibers with sarcolemma membrane attack complex deposition. Abundant MuRF-1 expression in IMNM and DM was associated with rapid activation of myogenesis after muscle injury, whereas relatively low expression of MuRF-1 in dysferlinopathy may be attributed to damaged muscle regeneration.

**Conclusions:**

MuRF-1 accumulated in regenerating myofibers, which may contribute to muscle injury repair in IMNM and DM. MuRF-1 staining may help clinicians differentiate IMNM and dysferlinopathy.

## Introduction

Immune-mediated necrotizing myopathy (IMNM) is a recently recognized idiopathic inflammatory myopathies (IIMs), featured by symmetrical proximal limb weakness and significantly enhanced creatine kinase (CK) (1). IMNM is further divided into three subtypes according to different serum autoantibodies, including anti-signal recognition particle (SRP)-positive IMNM, anti-3-hydroxy-3-methylglutaryl coenzyme A reductase (HMGCR)-positive IMNM and seronegative IMNM (1). To date, the molecular mechanisms of IMNM have yet to be elucidated.

Degradation of skeletal muscle proteins is regulated by four main proteolytic mechanisms, including autophagy-lysosome system, ubiquitin proteasome system (UPS), calcium-dependent calpains and caspases (2, 3). More recently, autophagy has been demonstrated to be involved in IMNM, and p62 (an autophagy marker) expression have been described as a consistent feature of IMNM (4–6). However, the role of the UPS in IMNM remains unclear. Activation of the UPS is a complex process, in which the muscle-specific E3 ubiquitin-ligase enzymes are responsible for specific recognition and binding to a target muscle protein (7). Muscle RING finger-1 (MuRF-1) is the most important E3 ligases and has been known as a muscle atrophy-related marker (3).

Muscle pathology in IMNM displays as prominent myofibers necrosis and regeneration, but no or few inflammatory infiltrates (4). Atrophic myofibers tend to occur in muscle biopsies from patients with a long disease duration, which is considered as irreversible (8, 9). A previous study has revealed that both anti-SRP and anti-HMGCR antibodies could induce myotubes atrophy and upregulate the expression of MuRF-1 *in vitro* (9). However, how MuRF-1 involved in IMNM is still unknown. Here we sought to investigate the expression of MuRF-1 and explore its role in IMNM.

## Materials and methods

### Patient selection

We enrolled 37 patients with IMNM treated at Tongji hospital from Jan 2017 to Feb 2022. Meanwhile, 13 dermatomyositis (DM) patients, 9 dysferlinopathy patients and 7 controls were selected for comparison. IMNM or DM was diagnosed based on the clinico-pathologic European Neuromuscular Center (ENMC) criteria followed by autoantibody testing (10, 11). Patients with dysferlinopathy were selected with confirmed genetic pathogenic variants in *DYSF* (*n* = 9). Controls were those who presented with non-specific muscle weakness or soft tissue complaints. Their serum CK levels were normal and no histological abnormalities were found on muscle biopsies after a series of staining, including hematoxylin-eosin (HE), modified Gömöri trichrome stain, nicotinamide adenosine dinucleotide, succinodehydrogenase, acid phosphatase, cyclooxygenase, periodic acid-schiff stain, sudan black, oil red O, adenosine triphosphatase PH 10.5/4.5/4.3 and immunostaining for major histocompatibility complex class I (MHC-I) and complement C5b-9/membrane attack complex (MAC).

All subjects except 2 patients with dysferlinopathy were tested for serum myositis-specific autoantibodies (MSAs) (anti-SRP, -HMGCR, -SAE, -Mi2, -MDA5, -TIF1γ, -NXP2, -JO-1, -EJ, -OJ, -PL-7, -PL-12,) and myositis-associated autoantibodies (MAAs) (anti-Ro52, -Ku, -PM-Scl 100, -PM-Scl 75). Several clinical and laboratory indexes at time of muscle biopsy were collected, such as blood routine, serum CK and lactate dehydrogenase (LDH) levels. Muscle strength was assessed using the manual muscle test (MMT)-8 (0–80) (12). Briefly, MMT-8 was tested unilaterally on a 0–10 scale with eight muscle groups, including 1 axial (neck flexor), 5 proximal (trapezius, deltoid, gluteus maximus, iliopsoas, and quadriceps), and 2 distal muscles (wrist flexor and ankle dorsiflexor). Grade 0 indicated no muscle contraction and grade 10 indicated normal power. The 8 muscle group subsets had a maximum potential score of 80.

### Muscle biopsies and immunostaining

Muscle biopsy was performed in all patients and specimens were well preserved at −80°C until use. Frozen tissues were sliced into 7 um sections for histological staining, immunohistochemistry (IHC) and immunofluorescence (IF) staining according to standard procedures as reported previously (13). The following primary antibodies were used to recognize: MuRF-1 (1:50, ab183094, Abcam), complement C5b-9/MAC (1:50, M0777, DAKO) and neural cell adhesion molecule (NCAM)/CD56 (1:50, ab6123, Abcam).

#### Reverse transcription-quantitative polymerase chain reaction (RT-qPCR)

Briefly, total RNA was extracted from biopsied muscle tissues using Trizol (Invitrogen). cDNA was synthesized using PrimeScript™ RT Master Mix (Perfect Real Time) (Takara). Afterward, PCR was performed using synthetic primers and SYBR gene PCR Master Mix (Yeasen). GAPDH was used as an internal reference. RT-qPCR reactions were performed with BioRad CFX Connect system. Gene expression were analyzed on the basis of the 2–ΔΔCT method. The primer sequences were as follows: MuRF-1, forward: 5′- TTTAGAGCACATAGCAGACGCC-3′, reverse: 5′-TTTAGAGCACATAGCAGACGCC-3′; GAPDH, forward: 5′- GGAGTCCACTGGCGTCTTCA-3′, reverse: 5′- GTCATGAGTCCTTCCACGATACC-3′.

### Statistical assessment

For further evaluation, five fields (original magnification ×200) were randomly selected from each section to count the total number of myofibers and the average percentages of myofibers positive for target antibody. Necrotic myofibers were defined as pale, coarse, hyalinized and/or phagocytised myofibers on H&E staining. Regenerating myofibers were defined as CD56 positive myofibers. CD56/NCAM has been known as one of the most useful stains for labeling regenerating fibers in clinical practice (4, 10), as its expression can be maintained during the proliferation and differentiation of myogenic cells (14). Image J software (NIH, Bethesda, USA) was used for slice analysis.

The differences of MuRF-1 expression between two groups were compared using Mann-Whitney *u*-test. Correlations among different variables were evaluated using Spearman's correlation coefficients. Statistical analysis was performed by GraphPad Prism 8.0. (Inc., La Jolla, CA, USA) and statistical significance was defined as *p* value < 0.05.

## Results

### Clinical and pathological data

Clinical and pathological data of all subjects are shown in Table 1. Of the 37 patients with IMNM, 27 patients (72.97%) had anti-SRP (*n* = 21) or -HMCGR (*n* = 6) antibodies, while the other 10 patients (27.03%) were seronegative. DM-associated MSAs were found in all 13 patients with DM, including anti-Mi-2 (*n* = 5), -NXP2 (*n* = 4), -MDA5 (*n* = 3) and -SAE1 (*n* = 1). No MSAs were found in dysferlinopathy patients or controls.

**Table 1:** Clinical and pathological data.

**Items**	**IMNM**	**DM**	**Dysferlinopathy**	**Controls**
Numbers	37	13	9	7
Age (years)^*^	46 (13–73)	49 (21–61)	32 (28–58)	35 (14–67)
Gender, female, *n* (%)	22 (59.46)	8 (61.54)	4 (44.44)	2 (28.57)
Course of disease, (months)^*^	5 (0.25–36)	2 (1–24)	24 (1–120)	7 (0.5–36)
MSAs positivity, *n* (%)	27 (72.97)	13 (100)	0 (0)	0
Not tested MSAs, *n* (%)	0 (0)	0 (0)	2 (22.22)	0
MMT-8^*^	62 (30–78)	63 (36–76)	77 (69–80)	80 (80–80)
Laboratory testing^*^				
WBC counts (10^9^ g/L)	7.05 (3.89–11.79)	6.36 (4.41–11.97)	5.72 (4.31–7.21)	5.87 (4.54–11)
Lymphocyte counts	1.66 (0.76–5)	1.08 (0.52–2.27)	1.81 (1.3–3.47)	1.70 (1.39–2.01)
CK (U/L)	5,206 (509–17,100)	1,387 (32–10,076)	6,325 (817–14,611)	68.5 (20–153)
LDH (U/L)	793 (201–2,712)	548 (130–1,876)	463 (194–839)	150.5 (132–209)
**Pathological findings**, ***n*** **(%)**				
Necrotic myofibers	34 (81.08)	10 (76.92)	7 (77.78)	0 (0)
Regenerating myofibers	37 (100)	13 (100)	9 (100)	0 (0)
MAC deposition on sarcolemma	15 (40.54)	4 (30.77)	1 (11.11)	0 (0)
MuRF-1 positive myofibers	35 (94.59)	13 (100)	9 (100)	0 (0)

CK, creatine kinase; DM, dermatomyositis; LDH, lactic dehydrogenase; *WBC*, white blood; LDH, lactic dehydrogenase; IMNM, immune-mediated necrotizing myopathy; MMT-8, manual muscle test 8 (0–80); MAC, membrane attack complex; MuRF-1, muscle RING finger-1.

* Data were shown by median and range, and obtained at the time of muscle biopsy.

### MuRF-1 staining is prominent in IMNM biopsy

**Figure 1:**
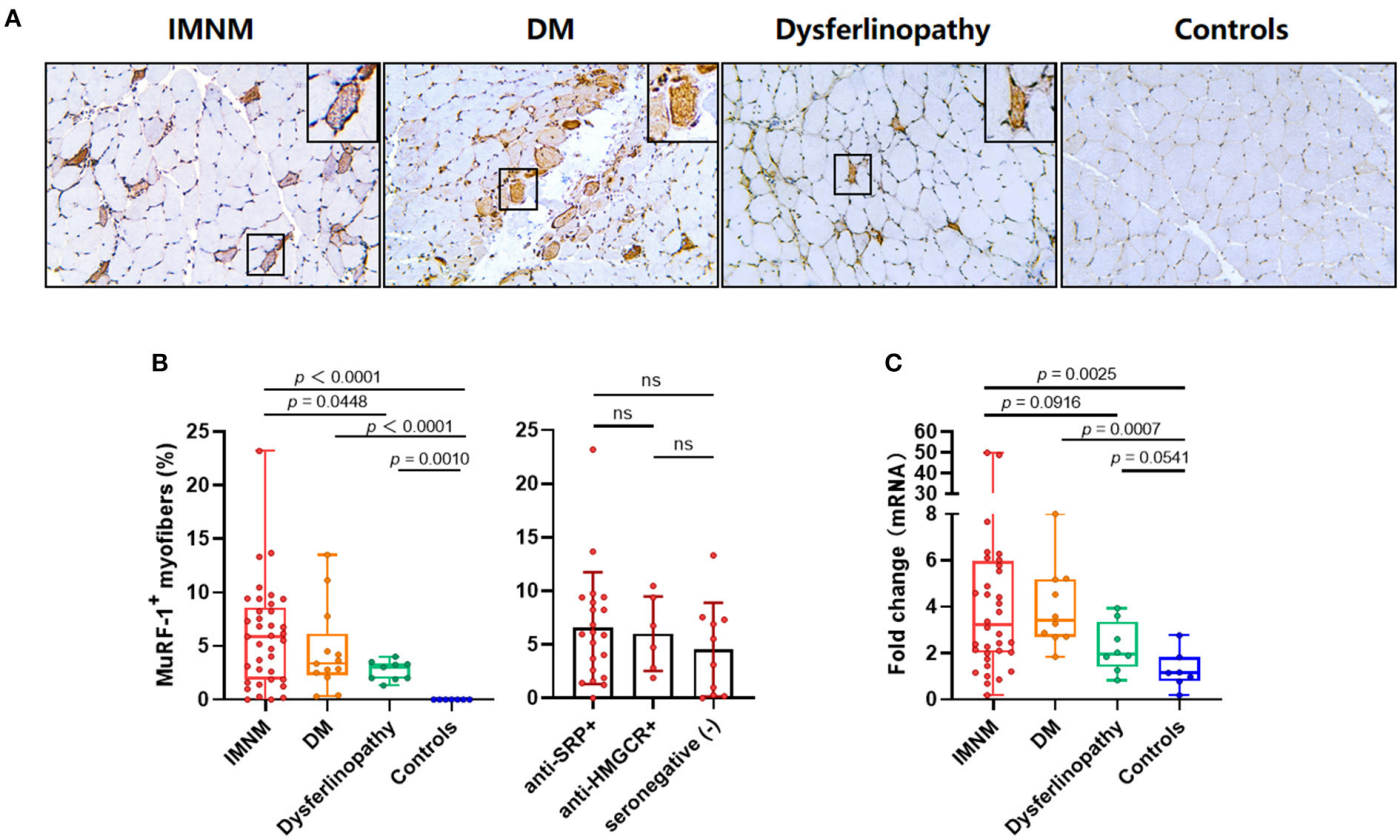
Staining pattern of muscle RING finger-1 (MuRF-1) in muscle biopsies. **(A)** Positive MuRF-1 staining in muscle biopsies from IMNM and dysferlinopathy with a diffuse distribution, as well as DM with a perifascicular distribution. No MuRF-1 staining was found in control biopsies. Magnification: ×200. **(B)** High percentage of MuRF-1 positive myofibers in IMNM biopsies. **(C)** The mRNA expression of MuRF-1 in muscle biopsies from IMNM, DM, dysferlinopathy and controls.

Strong MuRF-1 staining could be noted in muscle biopsies from IMNM, DM and dysferlinopathy, but consistently absent in controls (Figure 1A). MuRF-1 staining presented as a fine granular pattern throughout the sarcoplasm of myofibers, diffused randomly in muscles of IMNM and dysferlinopathy patients, and confined to the perifascicular area in muscles of DM patients (Figure 1A). Quantification revealed a high percentage of MuRF-1-positive myofibers in IMNM than in dysferlinopathy (*p* = 0.0448), but no difference between IMNM and DM, or anti-SRP+ and anti-HMGCR+ IMNM (Figure 1B). Similarly, the mRNA level of MuRF-1 was significantly upregulated in IMNM and DM, when compared with controls (*p* = 0.0025, *p* = 0.0007, respectively). MuRF-1 expression tended to be higher in IMNM than in dysferlinopathy, but was not statistically significant (*p* = 0.0916) (Figure 1C).

### The percentage of MuRF-1-positive myofibers correlates with muscle weakness and disease activity in IMNM and DM

**Figure 2:**
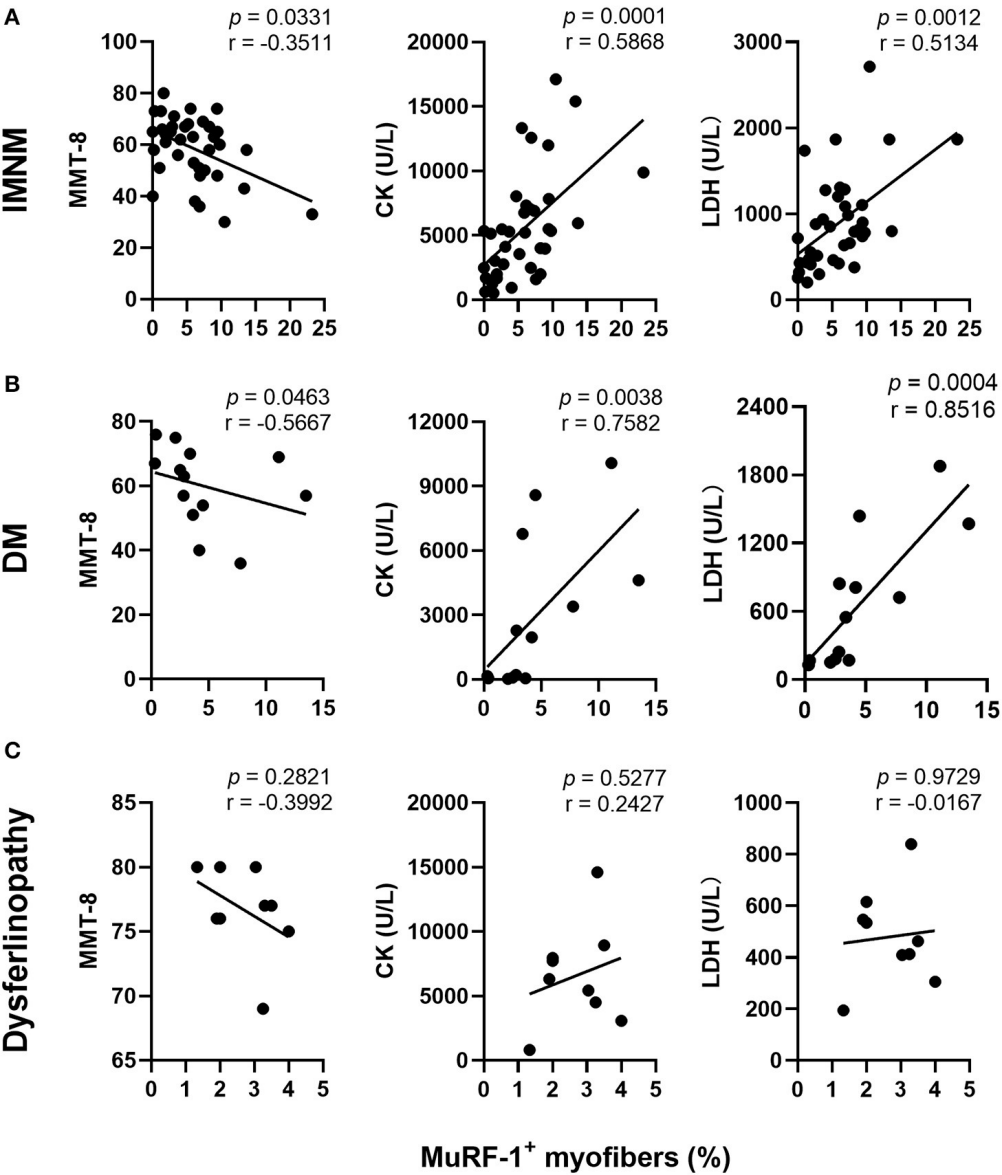
Correlations between the percentage of muscle RING finger-1 (MuRF-1) positive fibers and clinical severity of IMNM, DM and dysferlinopathy. **(A,B)** The percentage of MuRF-1-positive fibers in IMNM and DM was negatively correlated with manual muscle test 8 (MMT-8) scores, and positively correlated with serum creatine kinase (CK) or lactic dehydrogenase (LDH) levels at the time of biopsy. **(C)** The percentage of MuRF-1-positive fibers in dysferlinopathy had no significant correlations with MMT-8 scores, CK and LDH levels at the time of biopsy.

Muscle strength can be assessed using MMT-8 scores, while disease activity is usually evaluated using serum CK and LDH levels (markers of muscle damage). The percentage of MuRF-1-positive fibers was negatively correlated with MMT-8 scores, and positively correlated with serum CK or LDH levels in IMNM and DM (Figures 2A,B). There were no correlations between the percentage of MuRF-1-positive fibers and MMT-8 scores, CK and LDH levels in dysferlinopathy (Figure 2C). These results suggested that MuRF-1 expression positively correlates with muscle weakness and disease activity in IMNM and DM, but not in dysferlinopathy.

### MuRF-1 staining predominantly presents in regenerating myofibers but not in atrophic myofibers

**Figure 3:**
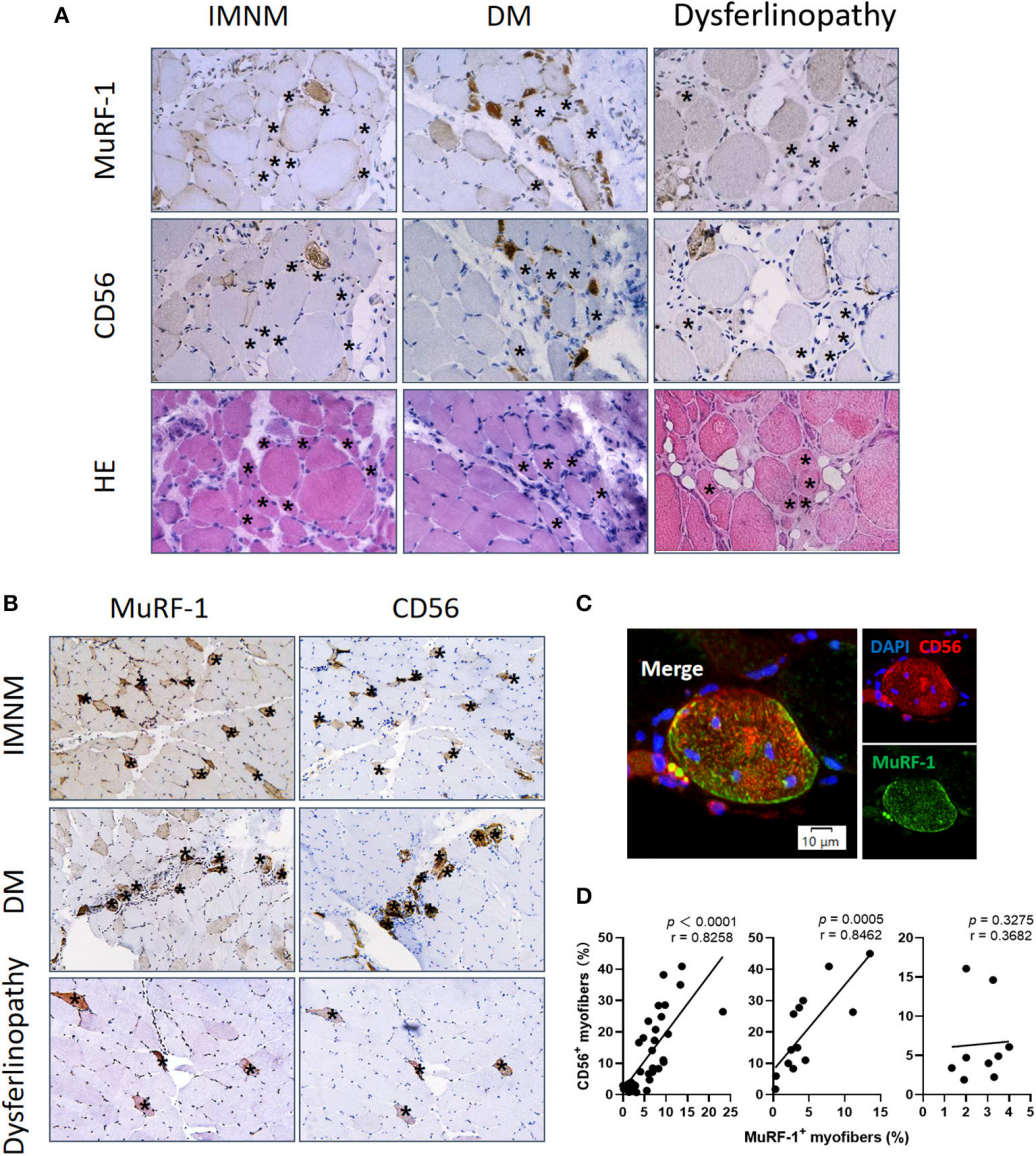
Muscle RING finger-1 (MuRF-1) accumulated in regenerating myofibers but not in atrophic myofibers. **(A)** MuRF-1 staining was absent in atrophic fibers (small fibers without CD56 staining and no basophilic appearance at H&E staining) (examples *). Magnification: ×400. **(B)** Serial-sections from IMNM, DM and dysferlinopathy biopsies showed most MuRF-1 positive myofibers stained by CD56 (examples *). Magnification: ×200. **(C)** Double immunofluorescence (IF) showed co-stained MuRF-1 and CD56 in the same myofiber in a IMNM biopsy. Scale bar, 10 μm. **(D)** Correlations between the percentage of MuRF-1 positive fibers and the percentage of CD56 positive fibers in IMNM, DM and dysferlinopathy.

Aiming to explore the effects of MuRF-1 on myofibers, muscle atrophy was first investigated. Muscle atrophy occurs as a consequence of increased muscle protein degradation (15). Atrophic myofibers (small fibers without CD56 staining and no basophilic appearance at H&E staining) predominantly presented in biopsies from dysferlinopathy with a diffuse distribution and DM with a perifascicular distribution (Figure 3A). For IMNM, atrophic myofibers were more likely to be found in biopsies from patients with a long course of disease (Figure 3A). Surprisingly, positive MuRF-1 staining was absent in atrophic fibers (Figure 3A), whereas many regenerating myofibers were detected to be positive for MuRF-1 (Figures 3B,C).

Consistently with MuRF-1-positive fibers, the distribution of regenerating myofibers was at random in IMNM and dysferlinopathy biopsies, whereas they primarily presented in perifascicular areas in DM biopsies (Figure 3B). Moreover, the percentage of MuRF-1-positive fibers was strongly correlated with the percentage of regenerating fibers in IMNM (*r* = 0.8258, *p* < 0.0001) and DM (*r* = 0.8462, *p* = 0.0005), while no significant correlation between them in dysferlinopathy was found (Figure 3D). Of note, MuRF-1 positivity was relatively common in early dysferlinopathy biopsies (Figure 3B), but was rarely seen in late biopsies (Supplementary Figure 1). Considering that dysferlinopathy is a progressive muscle wasting disease, this result strongly indicated the correlation between MuRF-1 expression and regenerative capacity rather than muscle atrophy. Moreover, most regenerating fibers in early dysferlinopathy biopsies were positive for MuRF-1 (Figure 3B), whereas only a few regenerating fibers were positive for MuRF-1 in late dysferlinopathy biopsies (Supplementary Figure 1). Therefore, it is reasonable to speculate that MuRF-1 may play a key role in the process of muscle regeneration in IMNM and DM, whereas muscle regeneration may be impaired in dysferlinopathy.

### MuRF-1-positive myofibers are distributed around damaged myofibers in IMNM and DM

Considering the correlations between MuRF-1 expression and disease severity in IMNM and DM, myonecrosis and complement deposits were then studied.

**Figure 4:**
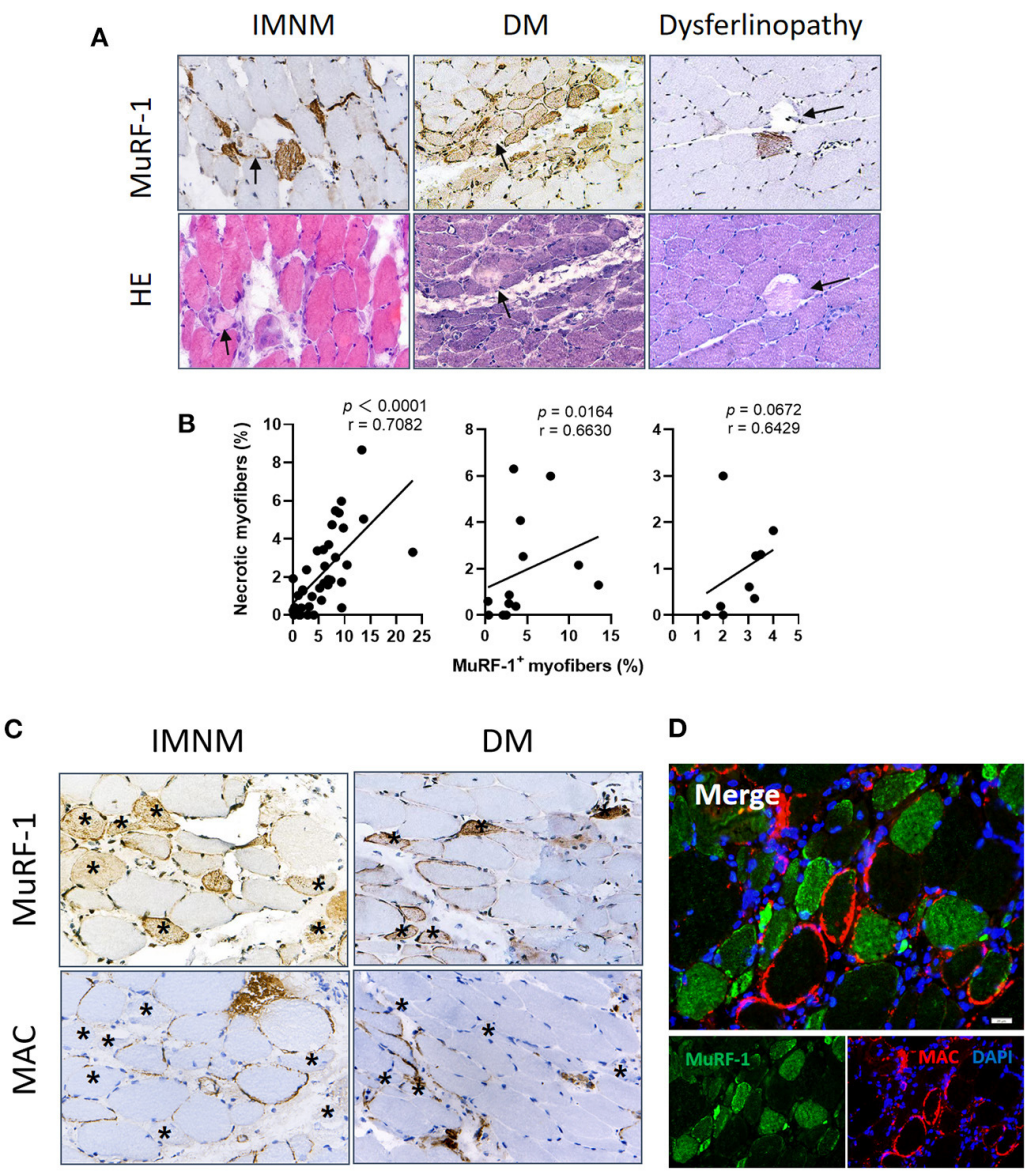
Muscle RING finger-1 (MuRF-1) staining were distributed around damaged myofibers. **(A)** Necrotic myofibers were negative for MuRF-1 in IMNM, DM and dysferlinopathy biopsies, whereas they were surround by MuRF-1-positive fibers (black arrows: necrotic fibers). Magnification: ×400. **(B)** Correlations between the percentage of MuRF-1 positive fibers and myonecrosis in IMNM, DM and LGMD. **(C)** Most myofibers with MAC staining were negative for MuRF-1 in IMNM and DM biopsies (examples *). Magnification: ×400. **(D)** Double immunofluorescence (IF) staining showed MuRF-1-positive myofibers distributed around myofibers with MAC deposition in a IMNM biopsy. Scale bar, 20 μm.

Myonecrosis is a non-specific pathological feature of multiple myopathies (16), which is prominent in IMNM and can be also found in DM and dysferlinopathy (Supplementary Figure 2). Necrotic myofibers were not stained positively for MuRF-1 in IMNM, DM and dysferlinopathy (Figure 4A). Inversely, MuRF-1-positive myofibers were distributed around necrotic myofibers (Figure 4A). Moreover, the proportion of MuRF-1-positive fibers was closely related to the percentage of myonecrosis in IMNM (*r* = 0.7082, *p* < 0.0001) and DM (*r* = 0.6630, *p* = 0.0164) (Figure 4B). There was a trend of weak association between them in dysferlinopathy (*r* = 0.6429, *p* = 0.0672) (Figure 4B).

Complement attacking on non-necrotic myofibers is an important pathological mechanism of myofibers damage in IIMs (4, 17). Sarcolemmic MAC deposition were analyzed, as its formation represents the final common pathway of complement activation. MuRF-1 positivity were only occasionally seen in myofibers with MAC deposition in IMNM and DM biopsies, whereas most myofibers positive for MuRF-1 staining were distributed around myofibers with MAC deposition (Figures 4C,D).

Considering the accumulation of MuRF-1 in regenerating fibers and its distribution pattern around damaged fibers, including necrotic fibers and non-necrotic fibers with MAC deposition, our results strongly suggested the involvement of MuRF-1 in muscle injury repair in IMNM and DM.

## Discussion

MuRF-1 plays a key role in mediating muscle proteins degradation (18, 19). Anti-SRP and anti-HMGCR antibodies can induce myotubes atrophy and upregulate the expression of MuRF-1 *in vitro* (9). In order to further explore the mechanism of MuRF-1 involved in IMNM, immunostaining was performed in biopsies from IMNM, DM, dysferlinopathy and controls. The results reveals that the expression of MuRF-1 is upregulated in IMNM, DM and dysferlinopathy compared to controls, and significantly correlated with muscle weakness and disease activity in IMNM and DM. Surprisingly, MuRF-1 staining is mainly observed in regenerating myofibers but not in atrophic myofibers. Furthermore, MuRF-1-positive myofibers tend to be distributed around necrotic myofibers and myofibers with sarcolemma MAC deposition in IMNM and DM. These findings suggest that MuRF-1 may participate in the process of myofiber regeneration, and contribute to muscle injury repair in IMNM and DM.

At the present study, positive staining of MuRF-1 was absent in atrophic myofibers in IMNM, DM and dysferlinopathy biopsies. This is inconsistent with previous findings demonstrating MuRF-1 as an atrophy-related marker (18, 19). Initial evidence for MuRF-1 as a marker of muscle atrophy was provided by transcriptome analysis that MuRF-1 was up-regulated in multiple models of atrophy, including denervation, immobilization, unweighting and glucocorticoid treatment (20). Moreover, mice deficient in MuRF-1 was found to be resistant to muscle atrophy after denervation (20). Several subsequent studies illustrated the key role of MuRF-1 in degrading contractile and structural proteins in myofibers, like titin, troponin 1 and myosin heavy chain (18, 19). Since then, more and more studies confirmed upregulated MuRF-1 in various conditions associated with muscle atrophy, such as sarcopenia of aging, disuse muscle atrophy, Cushing's syndrome, cancer cachexia and diabetes (3, 21–24). Of note, although the mRNA or protein levels of MuRF-1 have been shown to be upregulated, co-stain sections for MuRF-1 and some markers of regeneration or atrophy were always absent (3, 21–24). Moreover, these studies were performed primarily using animal or *in vitro* models, which may be different from real conditions in patients (3, 21–24). Therefore, it is uncertain whether MuRF-1 is actually involved in myofiber atrophy in patients with these conditions. Upregulated MuRF-1 expression in muscle tissues may be associated with regeneration, as muscle damage, including atrophy, is often accompanied by varying degrees of myofibers regeneration in real conditions, just like dysferlinopathy. Although the discrepancy between myositis and these models of skeletal muscle atrophy may be attributed to different pathogenetical background, MuRF-1 presenting as an atrophy-related marker obviously needs more evidence.

**Figure 5:**
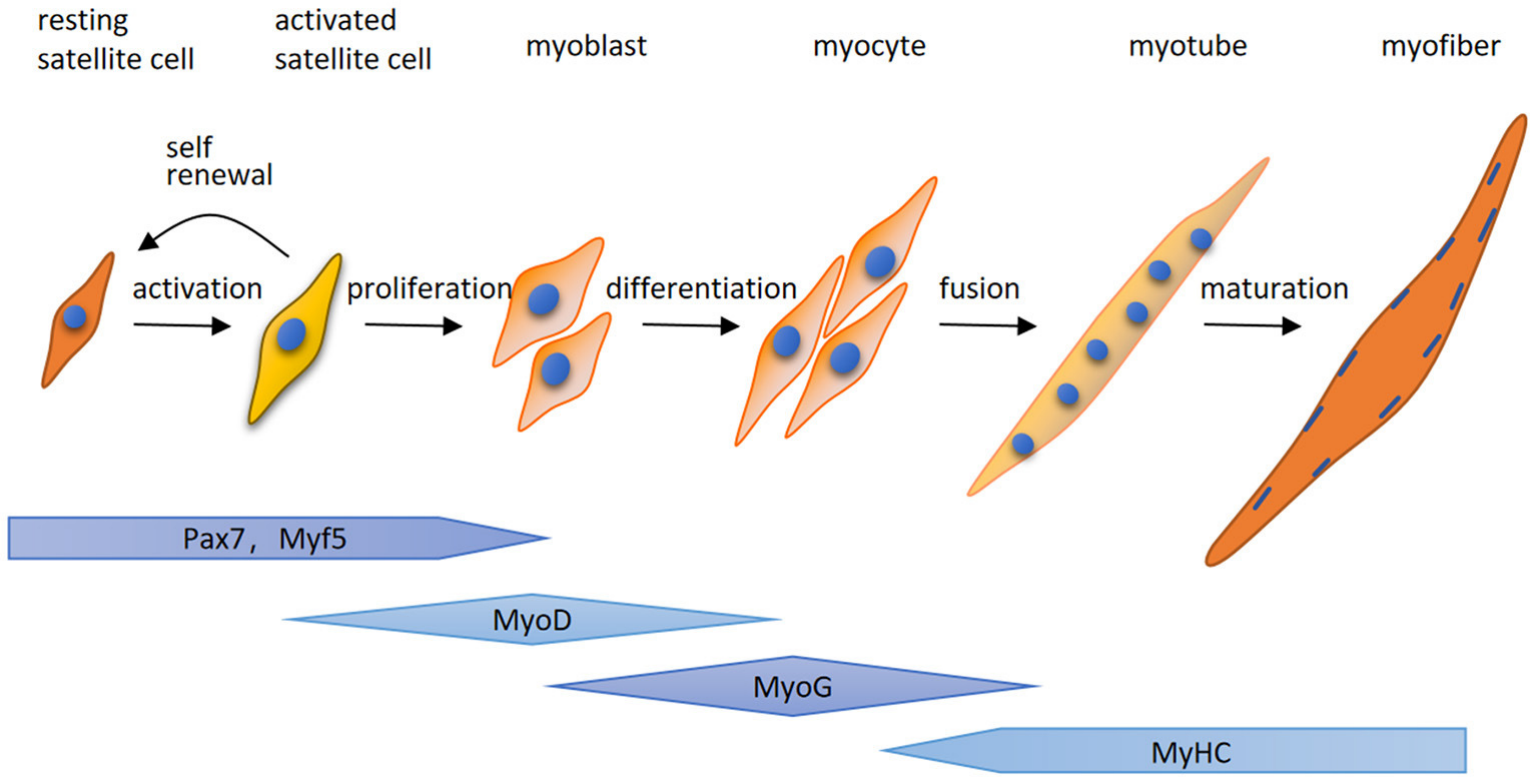
A schematic illustration of myogenesis. Quiet satellite cells are activated by various stimuli after muscle injury, undergo the proliferative phase and produce adequate myoblasts. After that, myoblasts differentiate into myocytes which fuse together to generate multinucleated myotubes and form mature myofibers. The process of myogenesis is highly controlled by sequential expression of paired box transcription factor paired box 7 (Pax7) and myogenic regulatory factors (MRFs), including myogenic regulatory factor 5 (Myf5), MyoD, myogenin (MyoG) and myosin heavy chain (MyHC).

Here for the first time, we highlight that MuRF-1 predominately accumulates in regenerating myofibers in human muscle tissues, which may help muscle injury repair in IMNM and DM. Regenerating fibers are morphologically similar to atrophic fibers due to their small size, however, their etiology and function is completely different (9). Muscle regeneration is a complex process, for which satellite cells are responsible (25). Satellite cells can be quickly activated by various stimuli after muscle injury and migrate to the damage area, where activated satellite cells start to expand, differentiate, fuse and form mature myofibers and eventually complete the repair of damaged skeletal muscle (Figure 5). The process of myogenesis is highly controlled by sequential expression of paired box transcription factor paired box 7 (Pax7) and myogenic regulatory factors (MRFs), including myogenic regulatory factor 5 (Myf5), MyoD, myogenin (MyoG) and myosin heavy chain (MyHC) (Figure 4) (26). To date, little insight into the roles of MuRF-1 in myogenesis is available. McElhinny and Gregorio et al. revealed that MuRF-1 can be expressed throughout muscle development, possibly playing a critical role in regulating myofibril turnover/assembly and muscle gene expression (27, 28). However, the specific mechanisms underlying MuRF-1 involved in myogenesis are still not well understood. The present study provides critical evidence for MuRF-1 involved in muscle regenerating, which actually deserves more attention and further investigation.

MuRF-1 contributes to muscle regeneration and correlates with myonecrosis, muscle weakness, and CK or LDH levels in IMNM and DM. It seems contradictory, but can be explained by rapid activation of myogenesis after muscle injury (myonecrosis or complement (MAC)-mediated muscle damage). In line with this, significant correlations were observed in IMNM between myonecrosis and muscle regeneration, serum CK and LDH levels, and MMT-8 scores (Supplementary Figure 3), which was also detected in a previous study (29). Similarly, significant correlations were also noted between the percentage of regenerating fibers and serum CK and LDH levels, and MMT-8 scores in IMNM (Supplementary Figure 4). No correlations between MuRF-1 expression with muscle regeneration, myonecrosis, muscle weakness and muscle damage in dysferlinopathy, suggest the impairment of regeneration. It was reported that *DYSF* gene was expressed in activated satellite cell and participated in myoblast fusion into myotube (30). Dysferlin deficiency directly affected myotube fusion and muscle regeneration (30, 31). Similarly, incapacity of Pax7-positive satellite cells to transit from proliferation to differentiation, eventually leads to damaged regeneration and fibrosis in limb-girdle dysferlinopathy muscular dystrophy (LGMD) R1 (31, 32). Besides dysferlinopathy and LGMD R1, satellite cell dysfunction seems to be a consistent pathological manifestation of many inherited neuromuscular conditions (31, 33). Ganassi et al. has defined those inherited neuromuscular diseases presenting as satellite cell dysfunction as “Satellite Cell-opathies” (31, 33). Therefore, it is concluded that gene mutations in dysferlinopathy lead to damaged muscle regeneration and a low expression of MuRF-1. Abundant MuRF-1 positive myofibers in IMNM and DM strongly indicate a better tissue repair ability, which is agreement with clinical observation. Dysferlinopathy is most frequently misdiagnosed as IIMs due to the similar clinicopathological features (4, 34). Immunostaining for MuRF-1 may help differentiate between dysferlinopathy and IIMs, especially seronegative IMNM.

There are some limitations. First, our study was performed in a single clinical center with a pretty limited sample size, which may have affected the statistical analysis. Second, even if the upregulation of MuRF-1 in IMNM and DM compared to controls has been confirmed by qPCR, a validation at protein level is still lack. Third, our study is limited by the observational and descriptive design based on muscle biopsies, therefore, the precise mechanisms of MuRF-1 involved in myogenesis cannot be determined. Regeneration as the physiologic consequence of necrosis, is important for muscle injury repair in IMNM (29). Although prominent co-distribution of regenerating fibers and MuRF-1 positive fibers was observed, we still can't rule out the possibility that MuRF-1 in regenerating fibers is a sign of aberrant repair, considering the negative correlation between MuRF-1 expression and MMT-8 score. Therefore, more studies are still needed to confirm our preliminary observation and further investigate the relevant mechanisms.

## Conclusions

Our study highlights the accumulation of MuRF-1 in regenerating myofibers but not in atrophic myofibers. Abundant MuRF-1-positive myofibers in IMNM and DM biopsies suggest a better tissue repair ability compared with dysferlinopathy. Immunostaining for MuRF-1 may be a useful tool to help clinicians differentiate between dysferlinopathy and IIMs, especially IMNM. More importantly, our finding will remind researchers that MuRF-1 presenting as an atrophy-related marker is questionable. More evidence, such as co-stain sections for MuRF-1 and markers of regeneration, should be provided, when upregulated MuRF-1 expression was observed in atrophic muscle tissues.

## Data availability statement

The original contributions presented in the study are included in the article/Supplementary material, further inquiries can be directed to the corresponding author.

## Ethics statement

The studies involving human participants were reviewed and approved by the Institutional Review Board at Tongji Hospital of Tongji Medical College, Huazhong University of Science and Technology. Written informed consent to participate in this study was provided by the participants' legal guardian/next of kin.

## Author contributions

M-GY contributed to the concept, design, and drafting of the manuscript. QZ, HW, XM, SJ, YL, and LX contributed to the acquisition, analysis, and interpretation of data. ZB contributed to the statistical analysis. BB has full access to all the data in the study, takes responsibility for the integrity of the data, the accuracy of the data analysis, contributed to the concept and design, and critical revision of the manuscript for important intellectual content. All authors contributed to manuscript revision, read, and approved the submitted version.

## Funding

This study was supported by the National Natural Science Foundation of China (Grant No. 81873758).

## Conflict of interest

The authors declare that the research was conducted in the absence of any commercial or financial relationships that could be construed as a potential conflict of interest.

## Publisher's note

All claims expressed in this article are solely those of the authors and do not necessarily represent those of their affiliated organizations, or those of the publisher, the editors and the reviewers. Any product that may be evaluated in this article, or claim that may be made by its manufacturer, is not guaranteed or endorsed by the publisher.
