# Supplementary figures and images for: The accumulation of muscle RING finger-1 in regenerating myofibers: Implications for muscle repair in immune-mediated necrotizing myopathy
Source: Front Neurol. 2022 Nov 24;13:1032738. doi: 10.3389/fneur.2022.1032738 (PMC9730696; doi:10.3389/fneur.2022.1032738)

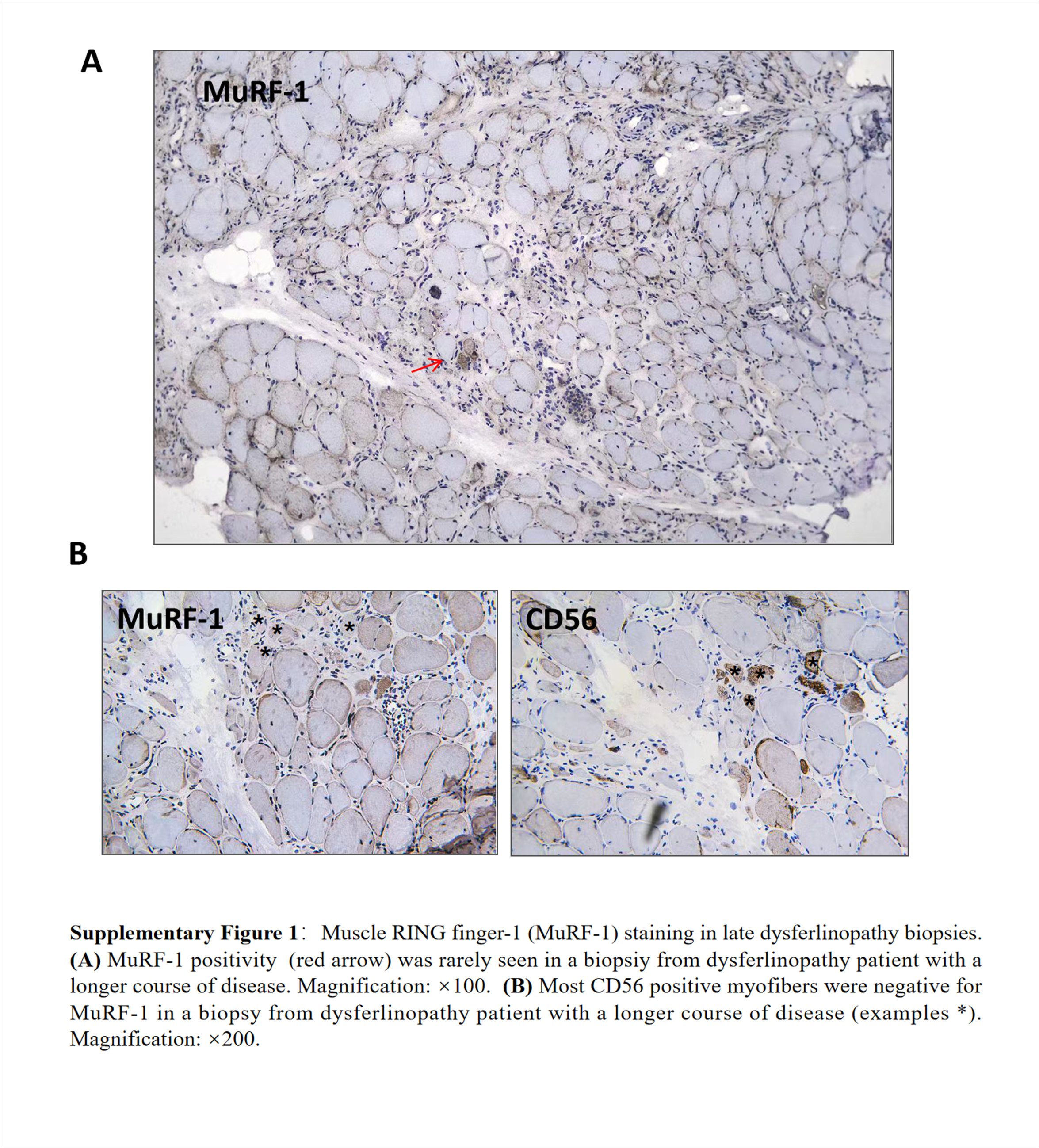

Supplement: Supplementary file 1 [file Image_1.TIF]

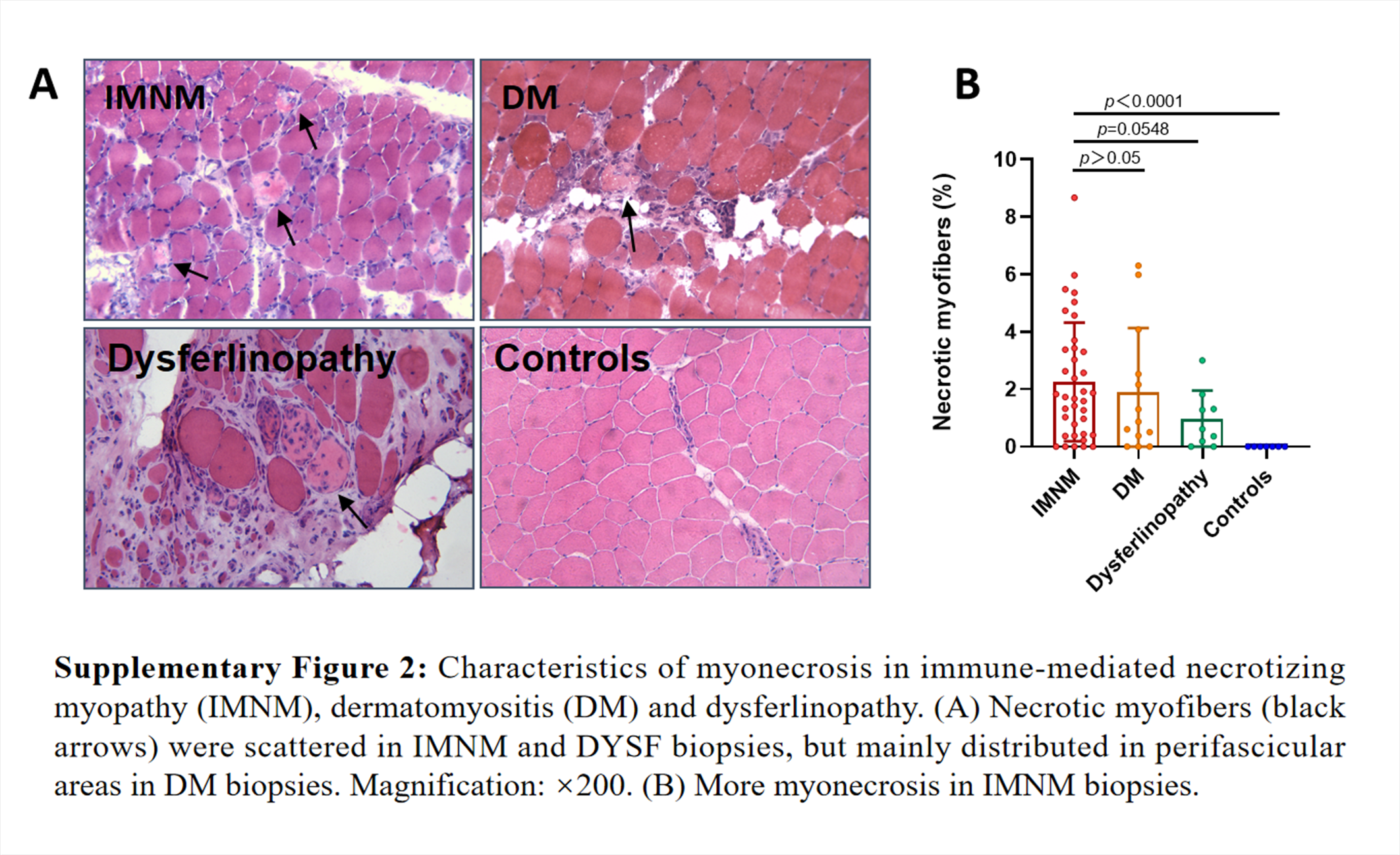

Supplement: Supplementary file 2 [file Image_2.TIF]

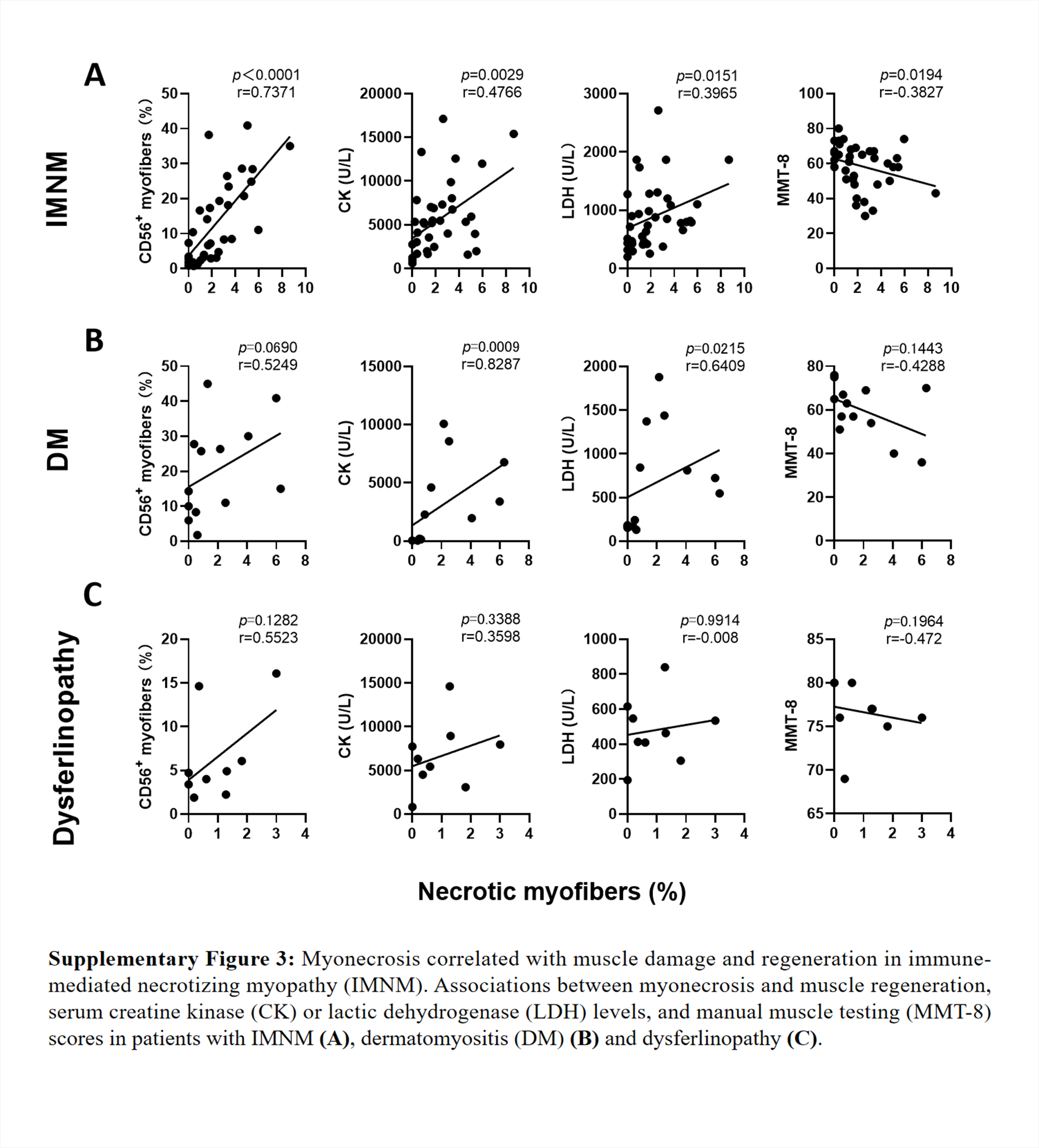

Supplement: Supplementary file 3 [file Image_3.TIF]

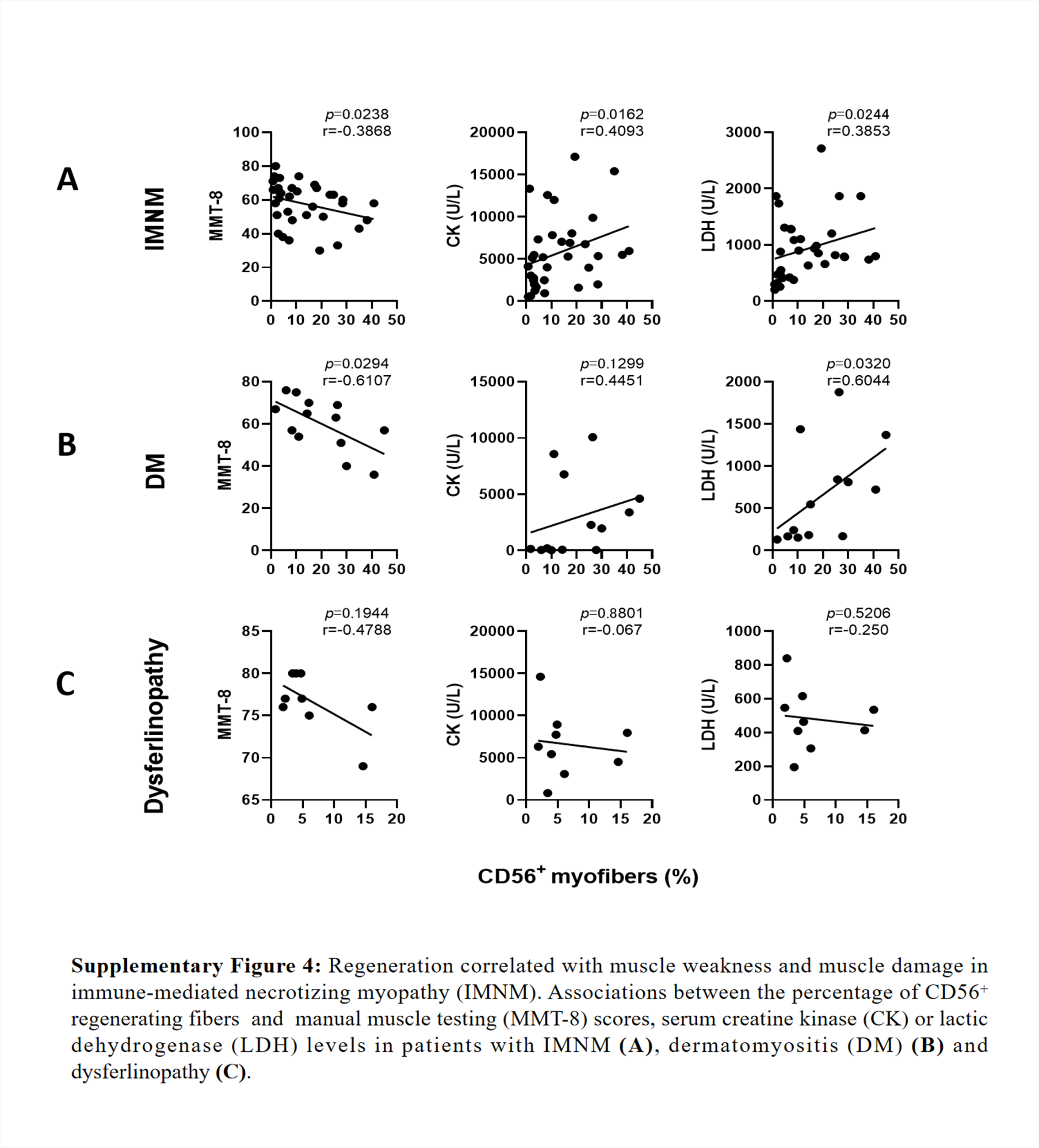

Supplement: Supplementary file 4 [file Image_4.TIF]
